# Chito-oligosaccharide composites enhanced the adaptability of cotton seedlings to salinized soil by modulating photosynthetic efficiency and metabolite

**DOI:** 10.3389/fpls.2025.1615321

**Published:** 2025-07-04

**Authors:** Mengjie An, Linlin Zhang, Qianqian Wang, Kaidi Ren, Qinjuan Wang, Dongmei Lin, Yongqi Zhu, Yonghong Fan

**Affiliations:** Xinjiang Key Laboratory of Biological Resources and Genetic Engineering, College of Life Science & Technology, Xinjiang University, Urumqi, Xinjiang, China

**Keywords:** composite soil amendment, salt stress, ion content, photosynthesis, carbohydrate metabolism

## Abstract

Agricultural production on salinized lands is an important direction of current agricultural research. Chito-oligosaccharide has been used as an excellent soil amendment in recent years. However, the mechanism of chito-oligosaccharide composites (COS-PA) impacting cotton seedlings on salinized lands is still unclear. In this study, the metabolic mechanism of COS-PA regulating cotton salt stress resistance was investigated by measuring seedling growth, leaf ion content, photosynthetic characteristics, and widely-targeted metabolic profiles. The results showed that salt stress reduced the contents of K^+^ and Ca^2+^ and enhanced the content of Na^+^ in cotton leaves compared to the control, which inhibited leaf photosynthesis and seedling growth. COS-PA application decreased leaf Na^+^ content significantly in salt-stressed cotton seedlings by 69.70%, and increased the leaf Ca^2+^ content, fresh weight of each plant part, transpiration rate, leaf chlorophyll concentration (Chl a), actual quantum yield, as well as stomatal conductance by 7.22%, 46.33%-96.36%, 96.65%, 44.53%, 27.15%, and 168.24%, respectively, compared with the no COS-PA application treatment. COS-PA application regulated the abundances of key leaf metabolites (L-lactic acid, Succinic acid, Methylmalonic acid, Aconitic acid, Citraconic acid), alleviating the salt stress. Therefore, COS-PA application could improve cotton seedling’s salt stress resistance by adjusting the growth characteristics, photosynthetic characteristics, and carbohydrate metabolism of cotton seedlings. The research will advance understanding of mechanisms by which COS-PA regulates the salt stress resistance of cotton seedlings and offer a scientific basis for salinized soil remediation and cotton yield improvement in arid areas.

## 1 Introduction

Soil salinization is an important factor restricting agricultural production as well as sustainable use of land resources in arid areas. About 6% of the world’s land (> eight hundred million hectares) is threatened by soil salinization (Jat Baloch et al., 2023). In China, there are about one hundred million hectares of salinized land, among them about eighty percent have not yet been undeveloped (Xie et al., 2021). Soil salinization threatens agricultural production as well as food security (Ondrasek and Rengel, 2021), and effective control of soil salinization is essential to improve crop yields. Because of the high salinity in soil parent material, low precipitation, and high evaporation in Xinjiang in western China, salinized cultivated lands are widely distributed, covering an area of 122.88 × 104 hm^2^, accounting for 37.72% of Xinjiang’s total arable land. Besides, the proportion is still on the rise. For a long time, soil salinization has become an important factor threatening local agriculture, environment, and sustainable development (Singh, 2021). Therefore, the development of new technologies for the efficient development and use of salinized lands is of great significance for sustainable agricultural production and regional development.

Chito-oligosaccharide is an alkaline oligosaccharide polymer composed of β-1,4 glycosidic bonds-linked glucosamine, and an important derivative for non-toxic natural polysaccharide chitosan (Kim and Rajapakse, 2005). Due to the superior characteristics such as high safety, high biocompatibility, and high degradability, it has been applied in agriculture (Guan et al., 2019). First of all, chito-oligosaccharide can activate the hydrolytic enzymes in plants. These enzymes play a pivotal role in decomposing and mobilizing the nutrients stored in seeds and other parts of plants (Turk, 2019). Secondly, low-molecular-weight chito-oligosaccharide are capable of stimulating cell division in plant roots. This is mainly achieved by activating key plant hormones such as auxin and cytokinin (Zou et al., 2016). In addition, chito-oligosaccharide not only exhibit a direct growth-promoting effect but also can serve as a biological inducer, thereby triggering the defense responses of plants (Yin et al., 2021). Many researches have shown that chitosan-oligosaccharide plays a positive role in various plant biological activities, including increasing nutrient absorption, photosynthetic rate, and stomatal conductance (G_H_2_O) of plants, inducing the synthesis of tobacco indoleacetic acid, and activating the antioxidant system in salt-stressed crops (Feng et al., 2019; Xue et al., 2022). In addition, foliar application with chitosan-oligosaccharide can also improve the immune resistance of rapeseed through the jasmonic acid/ethylene signaling pathway (Zong et al., 2017), as well as regulate the genes associated with plant defense response, carbon & nitrogen metabolism, photosynthesis, as well as transcription factors (Zhang et al., 2018). For instance, Zhang et al. (2017) showed that sprayed foliar application chitohexaose, chitoheptaose, and chitooctaose could promote the growth, improve the photosynthetic parameters, soluble sugar and proline content, then induce the expression of Na^+^/H^+^ antiporter genes of wheat seedling. Although it can improve crop growth activity, disease, drought, and salinity resistance (Liu et al., 2023), there are often multiple stresses in salinized soils, such as poor pore structure, soil nutrient scarcity, and high salt content, which need to be remediated from multiple directions. That is, the use of pure chitosan-oligosaccharide for salinized soil remediation is insufficient. Currently, the study on the synergistic effect of chito-oligosaccharide with trace elements, amino acids, and polymer materials is still lacking, especially the research on the combined use of multiple modifiers.

Cotton is an irreplaceable cash crop of Xinjiang, China, but cotton planting in Xinjiang is severely impacted by soil salinization currently. Continuous increase of soil salinity significantly reduces the emergence rate, leaf membrane permeability, photosynthetic characteristics, root growth (volume, surface area, length) of cotton (Yi et al., 2023), which ultimately leads to an reduction in the yield and quality (Sharif et al., 2019). Xie et al. (2022) reported that chito-oligosaccharide addition increased the expression of key genes as well as the activity of superoxide dismutase & NADPH oxidase in crops, reduced MDA content & cell membrane permeability, as well as regulated crop metabolism, thus increasing crop resistance, compared with the control group. However, the study of using only chito-oligosaccharides for the remediation of salinized lands is no longer sufficient to meet current agricultural production needs. Therefore, this study combined chitosan-oligosaccharides with trace elements, amino acids, and polymers to form COS-PA, which was then applied to salinized cotton fields to analyze the changes in the growth, development, and metabolic levels of cotton seedlings. The objectives were to clarify (1) whether exogenous application of COS-PA could alleviate the damages of salt stress to photosynthesis & physiology of cotton seedlings and (2) the metabolic mechanism of COS-PA regulating cotton seedlings resistance to salt stress. This study will provide ideas for the remediation & utilization of salinized lands.

## 2 Materials and methods

### 2.1 COS-PA characteristics

In this study, based on chito-oligosaccharide, manganese sulfate, amino acid complex, γ-polyglutamic acid, polyaluminium chloride, magnesium sulfate, and calcium monophosphate were used to prepare a new chito-oligosaccharide modifier COS-PA. The specific procedures were as follows:

(1) Deionized water (1 L) was heated to 40-45°C, and then mixed with 1–3 g of citric acid in a flat-bottomed flask on a magnetic stirrer. After stirring, 100 g of chito-oligosaccharides were added, stirred for 2–3 h, to obtain mixture A (pH: 4.8). (2) Manganese sulfate (10–15 g) was added to the mixture A. After cooling to 25-35°C, the mixture was stirred until completely dissolved, to obtain mixture B (pH: 4.8). (3) Amino acid complex (10–15 g) and γ-polyglutamic acid (5–10 g) were added to the mixture B, heated to 50-60°C, and stirred for 0.5–1 h until completely dissolved, to obtain mixture C (pH: 4.8). (4) Polyaluminium chloride (5–10 g) was mixed with mixture C, followed by 12–15 h stirring while keeping the temperature unchanged, to obtain mixture D (pH: 4.7). (5) Anhydrous magnesium sulfate (10–15 g) was mixed with mixture D, heated to 65-75°C, stirred for 0.5–1 h until completely dissolved, to obtain mixture E (pH: 4.7). (6) Calcium monophosphate (5–10 g) was mixed with mixture E, followed by 5–8 h stirring while the temperature was kept unchanged, to obtain mixture F (pH: 4.6). (7) After 24 hours, the mixture F was centrifuged (Thermo Fisher, USA) at 4000 r for 10 min and filtered for 10 min. The precipitation was discarded, and the supernatant (a new chito-oligosaccharide modifier COS-PA) was transferred into a bottle and sealed.

### 2.2 Experimental design

This indoor pot trial was carried out in May 2024 in the Plant Cultivation Room, Xinjiang University, China. For the potted plants, the ambient temperature ranged from 24°C-28°C. The light regime was set at 16 h (light)/8 h (darkness), with a light intensity of 3000 lx. The relative humidity was maintained at 70%-75%. A total of four treatments were designed, namely (1) CK, no salt stress and no COS-PA application; (2) COS-P treatment, no salt stress but 100 mg·L^-1^ of COS-PA was applied; (3) SA treatment, salt stress (salt concentration: 10 g·L^-^1) but no COS-PA application; (4) CP treatment, salt stress (salt concentration: 10 g·L^-^1) plus 100 mg·L^-1^ of COS-PA application. Each treatment had three replicates/pots (height: 25 cm; diameter: 20 cm). The salts for salt stress were prepared as follows: NaCl, Na_2_SO_4_, NaHCO_3_, and NaCO_3_ were mixed according to the ratio of 12: 9: 8: 1 (molar ratio). Then, 25.0 g of the mixed salts was dissolved in 2.5 L of distilled water, and added to the pots according to the mass ratio of 1: 1 (water: mixed salts) (Wang et al., 2021b).

Cotton seeds (Xinluzao 72) were subjected to 5-min disinfection with 70% alcohol, three times of rinsing with sterile water, and 10–15 min soaking in 3% hydrogen peroxide solution. Vigor seeds were selected and 40 seeds were sown in each pot in May 2024. After the seedlings had two true leaves, 3 plants were retained in each pot. Insecticide (imidacloprid) was sprayed every three days. Irrigation was conducted every three days using deionized water, maintaining a stable water-holding capacity (60%). Seedlings were fertilized every seven days (0.077 g of urea, 0.128 g of diammonium, and 0.026 g of potassium sulfate per 1 kg of soil). Mixed salt solution and COS-PA were added on the second day after extra seedling removing, specifically, 100 mL of the salt solution was added every day for five consecutive days, and a total of 500 mL was added in each pot. This approach aimed to stabilize the salt ion in the soil, thereby avoiding salt shock. Meanwhile, to ensure the salt solution concentration remained constant across all treatments, the pots were weighed and water was replenished twice a day. The concentration of COS-PA was 100 g·L^-1^, and 100 mL of COS-PA solution was added every day for three consecutive days. The staged application of COS-PA was primarily intended to facilitate the slow release of its major components. This is beneficial for extending the control effect of COS-PA on salt ions. Root fresh weight, leaf fresh weight, and plant height were determined after 60 days. After that, one part of the fresh leaves were used for the determination of K^+^, Na^+^, Ca^2+^ contents and photosynthetic characteristics of cotton, and the other part was used for the determination of metabolomics.

### 2.3 Determination of cotton seedling growth index and ion content

The stem thickness & plant height of cotton seedlings (three seedlings per treatment) were measured using vernier caliper and ruler, respectively. After absorbing the surface moisture of cotton seedlings, the fresh weight of leaves, stems, roots, and total weight were measured using an electronic balance.

The contents of K^+^, Na^+^, Ca^2+^, and physiological parameters (photosynthetic parameters & chlorophyll content) of cotton seedlings (three seedlings per treatment) were measured, followed by the calculation of the average values. Specifically, leaf samples were pre-processed by nitric acid-perchloric acid mixture digestion method, and the Ca^2+^, Na^+^, and K^+^ contents of cotton seedling leaves were measured by ICP-AES (Optima-7000DV, PE, USA) (Ren et al., 2024).

### 2.4 Determination of photosynthetic characteristics of cotton seedlings

The intercellular carbon dioxide concentration (*Ci*), net photosynthetic rate (*A*), *G_H_2_O*, photosynthetic rate, as well as transpiration rate (*E*) of cotton seedlings (three seedlings per treatment) were measured by a photosynthetic instrument (WLZG-FS-3000, Heinz Walz GmbH, German). Each leaf was measured three times (Grinberg et al., 2022).

Then, the leaves were subjected to dark adaption, to determine the maximum optical quantum yield (*Fv/Fm*), maximum fluorescence (*Fm*), and initial fluorescence (*F0*) using the chlorophyll fluorescence imaging system. Three plants were measured for each treatment. Then, the non-regulated energy dissipation *Y(NO)*, regulated energy dissipation *Y(NPQ)*, effective quantum efffciencies of PSII (*Y(II)*), and maximum quantum yield (*Fv/Fm*) were computed (Zhao et al., 2024).

Cotton seedling leaves were extracted with 95% ethanol solution for 24 hours in dark. Absorbance values were then measured with a spectrophotometer (Shandong Ouleibo Instrument Co., LTD, China) at 470 nm (OD470), 649 (OD649), and 665 (OD665). Based on the following formulas (Wang et al., 2024), the Chla concentration (Equation 1), Chlb concentration (Equation 2), Chla/Chlb ratio, as well as total chlorophyll concentration (Chl) (Equation 3) were computed:


(1) $$\text{Chl a }\left(\text{mg}\cdot {\text{L}}^{-1}\right)=13.95\text{ OD}665-6.88\text{ OD}649$$

(2) $$\text{Chl b }\left(\text{mg}\cdot {\text{L}}^{-1}\right)=24.96\text{ OD}649-7.32\text{ OD}665$$

(3) $$\text{Chl }\left(\text{mg}\cdot {\text{g}}^{-1}\text{ FW}\right)=\left(\text{C}\times \text{V}\right)/\left(1000\times \text{W}\right)$$

where C denotes pigment concentration in extraction solution, V denotes extraction solution volume, and W denotes sample mass.

### 2.5 Metabolome analysis

After 60 days of cultivation, three true leaves were selected in each treatment and sent to Beijing Biotech Biotechnology Co., Ltd, China. After rinsing, cutting into sections, and frozing in liquid N, 100 mg of leaf samples were ground in liquid N, followed by addition of methanol aqueous solution (80%, 500 μL), vortexing, and ice bath for five minutes. Then, the samples were subjected to a 20-min centrifugation (15,000 g, 4°C), and the supernatant was diluted using water to make the methanol content reach 53%. After another 20-min centrifugation (15,000 g, 4°C), the supernatant was collected for following measurements. Equivalent-volume QC (Quality control) samples were prepared through sample mixing, and sample blank solution was prepared using methanol aqueous solution (53%) (Li et al., 2020).

A chromatograph and a chromatographic column were used for chromatography, with the flow rate of 0.2 mL/min and the column temperature of 40°C. For the negative mode, 5 mmol/L ammonium acetate (pH: 9.0) and methanol were used as the mobile phase A and B, respectively. For the positive mode, 0.1% formic acid and methanol were used as the mobile phase A and B, respectively. A mass spectrometer was used for mass spectrometry, with the scan range of 100–1500 m/z, the aux gas heater temperature of 350°C, the S-lens RF level of 60, the capillary temperature of 320°C, the aux gas flow rate of 10 L/min, the sheath gas flow rate of 35 psi, and the spray voltage of 3.5 kV. To further understand the interaction of DAMs, the KEGG database was used to annotate DAMs.

### 2.6 Statistical analysis

Duncan’s test, one-way ANOVA, and statistical analysis were carried out on growth parameters, ion content, and photosynthetic parameters of cotton seedlings in Origin 8.0 & SPSS 25.0 (*p<* 0.05). Charts were drawn in Origin 8.0 and Adobe Illustrator CS6 (Adobe, USA). KEGG pathways and PCA results were plotted with “ggplot2” package in R 3.6.1. Mantel test was conducted with “linkET” package for clarifying the correlations between key DAMs and cotton seedling growth indexes, ion content, and photosynthetic characteristics under different treatments.

## 3 Results

### 3.1 Analysis of cotton seedling growth characteristics and ion content

**Figure 1:**
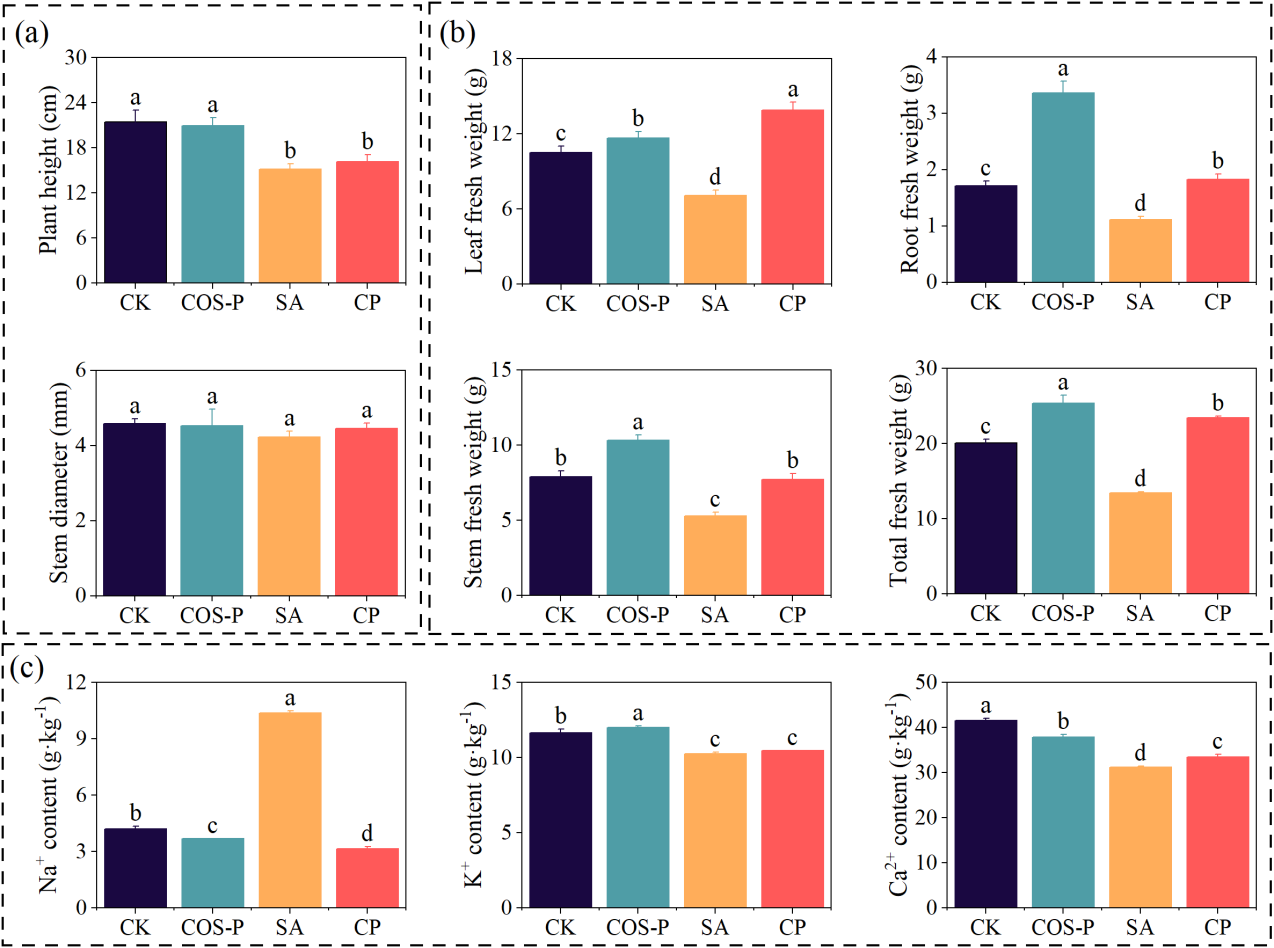
Growth indexes **(a, b)** and Na^+^, K^+^, Ca^2+^ contents **(c)** of cotton seedlings under different treatments. CK, no salt stress and no COS-PA application; COS-P, no salt stress but 100 mg·L^-1^ of COS-PA was applied; SA, salt stress (10 g·L^-1^ of salts) but no COS-PA application; CP, salt stress (10 g·L^-1^ of salts) and 100 mg·L^-1^ of COS-PA application. The same below. Different lowercase letters indicate significant difference between treatments (*p< 0.05*).

COS-P significantly regulated salt-stressed cotton growth and ion homeostasis (**Figure 1**). The plant height of cotton seedlings of the SA and CP treatments reduced by 29.50% and 24.77%, respectively compared to CK (*p<* 0.05) (**Figure 1a**), and COS-P, SA, and CP treatments had no effect on the stem diameter of cotton seedlings (**Figure 1a**). The leaf, stem, root, and total fresh weight of the COS-P treatment increased (*p<* 0.05) by 11.06%, 30.45%, 96.44%, and 25.95%, respectively, and those of SA treatment reduced (*p<* 0.05) by 32.66%, 33.22%, 34.87%, and 33.06%, respectively (**Figure 1b**), compared to CK. The leaf, stem, root, as well as total fresh weight of CP treatment enhanced (*p<* 0.05) by 96.36%, 46.33%, 64.13%, and 74.06%, respectively compared to SA treatment (**Figure 1b**). The leaf Na^+^ & Ca^2+^ contents of COS-P treatment reduced (*p<* 0.05) by 12.43% and 8.95%, respectively, while leaf K^+^ content enhanced by 2.85% (*p<* 0.05), compared with CK. The leaf Na^+^ content of the SA treatment enhanced (*p<* 0.05) by 146.42%, and the leaf K^+^ and Ca^2+^ content reduced (*p<* 0.05) by 11.96% and 24.80%, respectively, compared to CK (**Figure 1c**). The Na^+^ content in the CP treatment reduced (*p<* 0.05) by 69.70% and the Ca^2+^ content increased by 7.22% compared to SA (**Figure 1c**).

### 3.2 Analysis of photosynthetic characteristics of cotton seedling leaves

**Figure 2:**
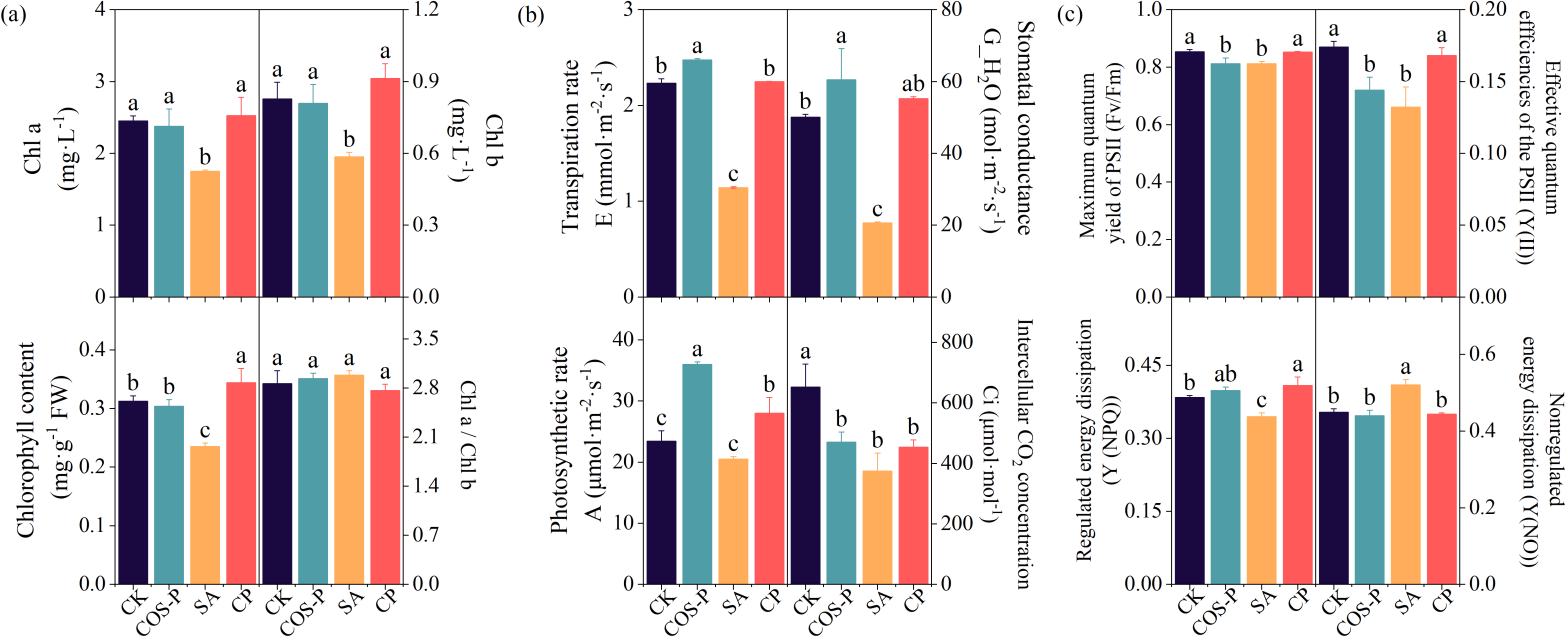
Photosynthetic parameters of cotton seedling leaves under different treatments. **(a)** Photosynthetic pigment content; **(b)** Photosynthetic performance; **(c)** Chlorophyll fluorescence parameters. Different lowercase letters indicate significant difference between treatments (*p< 0.05*).

COS-PA significantly increased the photosynthetic rate, chlorophyll fluorescence parameters, and photosynthetic pigments in salt-stressed cotton seedlings compared with CK (**Figure 2**). The leaf Chl, Chl b, and Chl a contents of the SA treatment reduced (*p<* 0.05) by 24.78%, 29.23%, and 28.67%, respectively compared to CK. The leaf Chl, Chl b, and Chl a contents of CP treatment increased (*p<* 0.05) by 46.31%, 55.83%, and 44.53%, respectively compared to SA (**Figure 2a**).

The leaf *A*, *G_H_2_O*, and *E* of the COS-P treatment enhanced by 53.51%, 20.81%, and 10.96%, respectively, and the *Ci* decreased by 27.89%, compared with those of CK. The leaf *E, G_H_2_O*, and *A* of the SA treatment reduced (*p<* 0.05) by 48.75%, 58.82%, and 42.51%, respectively compared to CK. The *E*, *G_H_2_O*, as well as *A* of the CP treatment increased (*p<* 0.05) by 96.65%, 168.24%, and 36.63%, respectively compared to SA treatment (**Figure 2b**).

The leaf *Fv/Fm* as well as *Y(II)* of COS-P treatment reduced (*p<* 0.05) by 4.91% and 4.87%, respectively, and the leaf *Y(II)*, *Y(NPQ)*, and *Fv/Fm* of the SA treatment reduced (*p<* 0.05) by 24.09%, 10.26%, and 17.21%, respectively, compared to CK. The leaf *Fv/Fm*, *Y(II)*, and *Y(NPQ)* of the CP treatment increased by 5.01%, 27.15%, and 18.69%, respectively, and the leaf *Y(NO)* decreased (*p< 0.05*) by 14.64%, compared to SA treatment (**Figure 2c**).

### 3.3 Analysis of metabolites in cotton seedling leaves

**Figure 3:**
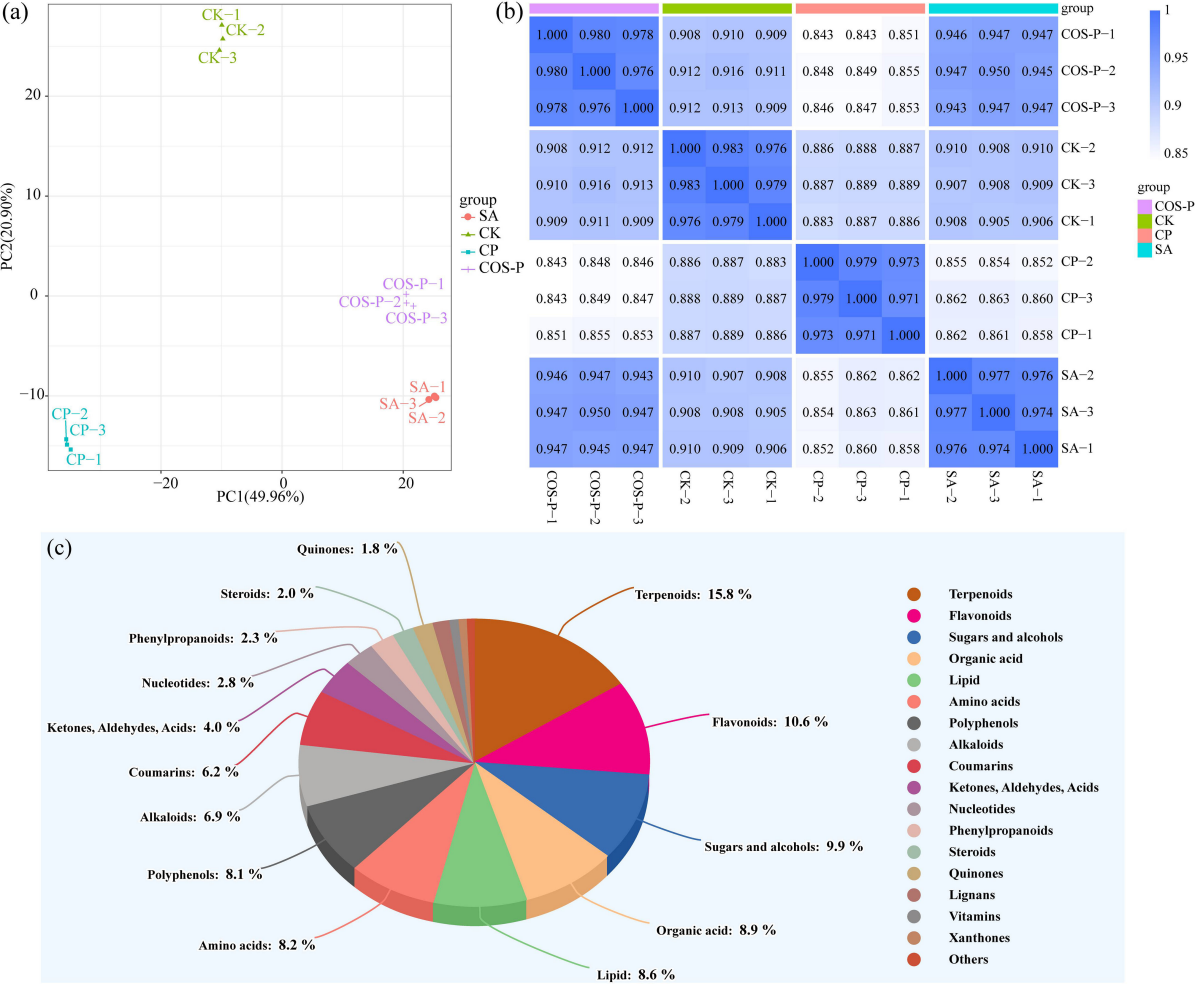
Principal component analysis (PCA) **(a)**, correlation analysis **(b)**, and classification **(c)** of metabolites.

**Figure 3** shows the quality evaluation of metabolomic data of cotton seedling leaves. PCA results showed that PC1 and PC2 explained 49.96% and 20.90% of the total variation, respectively. The PC1 axis clearly separated the CK and CP treatments from the SA and COS-P treatments, and the PC2 axis clearly separated the CK from the SA and CP treatments (**Figure 3a**). In addition, correlation analysis showed a high degree of similarity between samples within each group and a great variation between groups (**Figure 3b**). There were 1,303 metabolites for the samples, which could be classified in 18 classes. Terpenoids were the most abundant metabolites in cotton seedling leaves, accounting for 15.8%, followed by Flavonoids (10.6%), Sugars and alcohobs (9.9%), Organic acid (8.9%), Lipid (8.6%), Amino acids (8.2%), and Polyphenols (8.1%) (**Figure 3c**).

**Figure 4:**
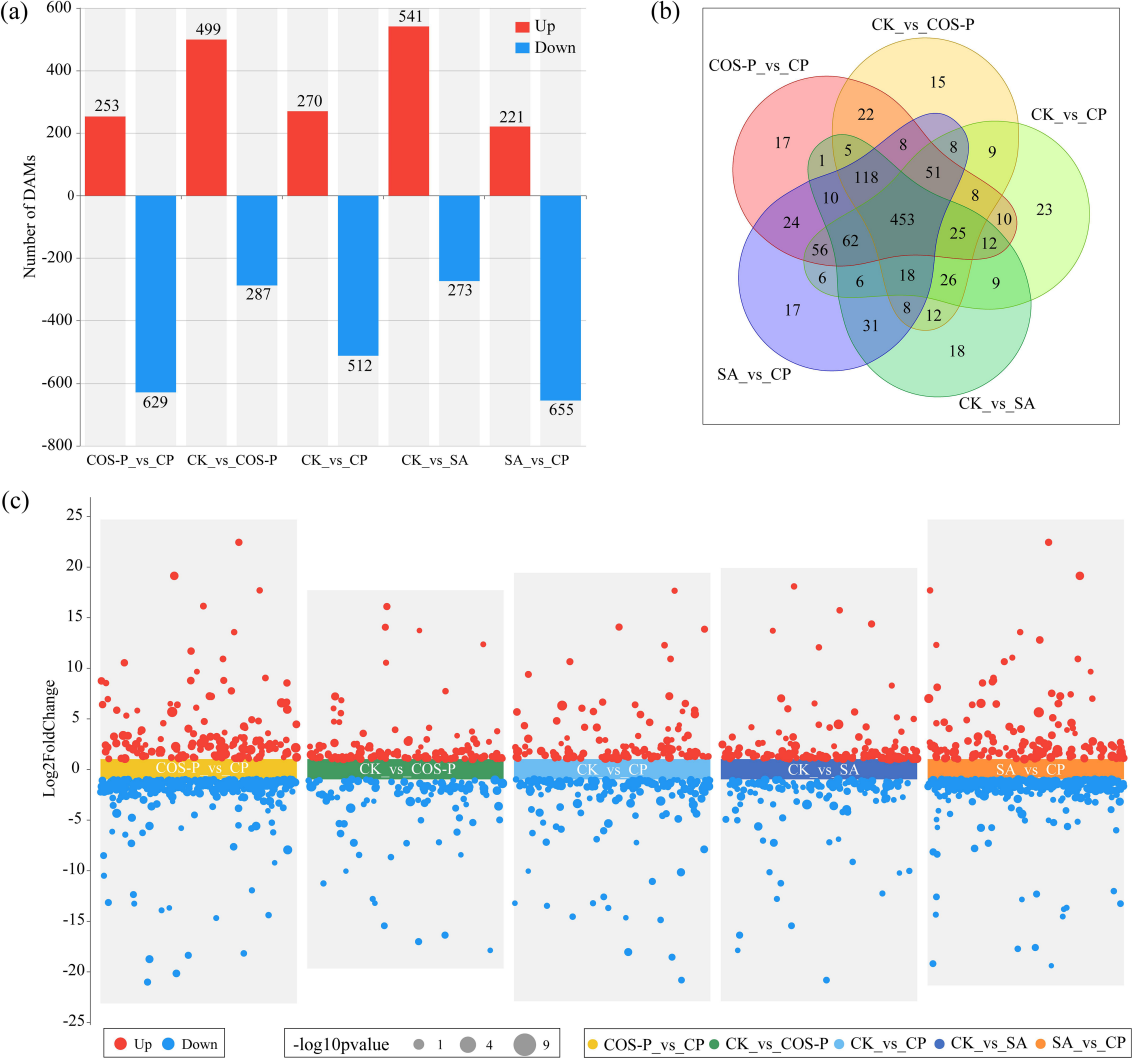
Differentially abundant metabolites (DAMs) in cotton seedlings leaves. **(a)** Number of DAMs; **(b)** Venn diagrams of DAMs; **(c)** Statistics of the up- and down-regulated DAMs in pairwise comparison of treatments.

**Figure 4** shows the differentially abundant metabolites (DAMs) in cotton seedling leaves. A total of 882 DAMs were identified in CP *vs* COS-P (253 up-regulated metabolites; 629 down-regulated metabolites). Seven hundred and eighty-six DAMs were identified in COS-P *vs* CK (499 up-regulated metabolites; 287 down-regulated metabolites). Seven hundred and eighty-two DAMs were identified in CP *vs* CK (270 up-regulated metabolites; 512 down-regulated metabolites). Eight hundred and fourteen DAMs were identified in SA *vs* CK (541 up-regulated metabolites; 273 down-regulated metabolites). Eight hundred and seventy-six DAMs were identified in CP *vs* SA (221 up-regulated metabolites; 655 down-regulated metabolites) (**Figures 4a, c**). According to the Venn diagram, 453 DAMs were identified in the four treatments (**Figure 4b**).

### 3.4 Analysis of carbohydrate metabolism of cotton seedling leaves

**Figure 5:**
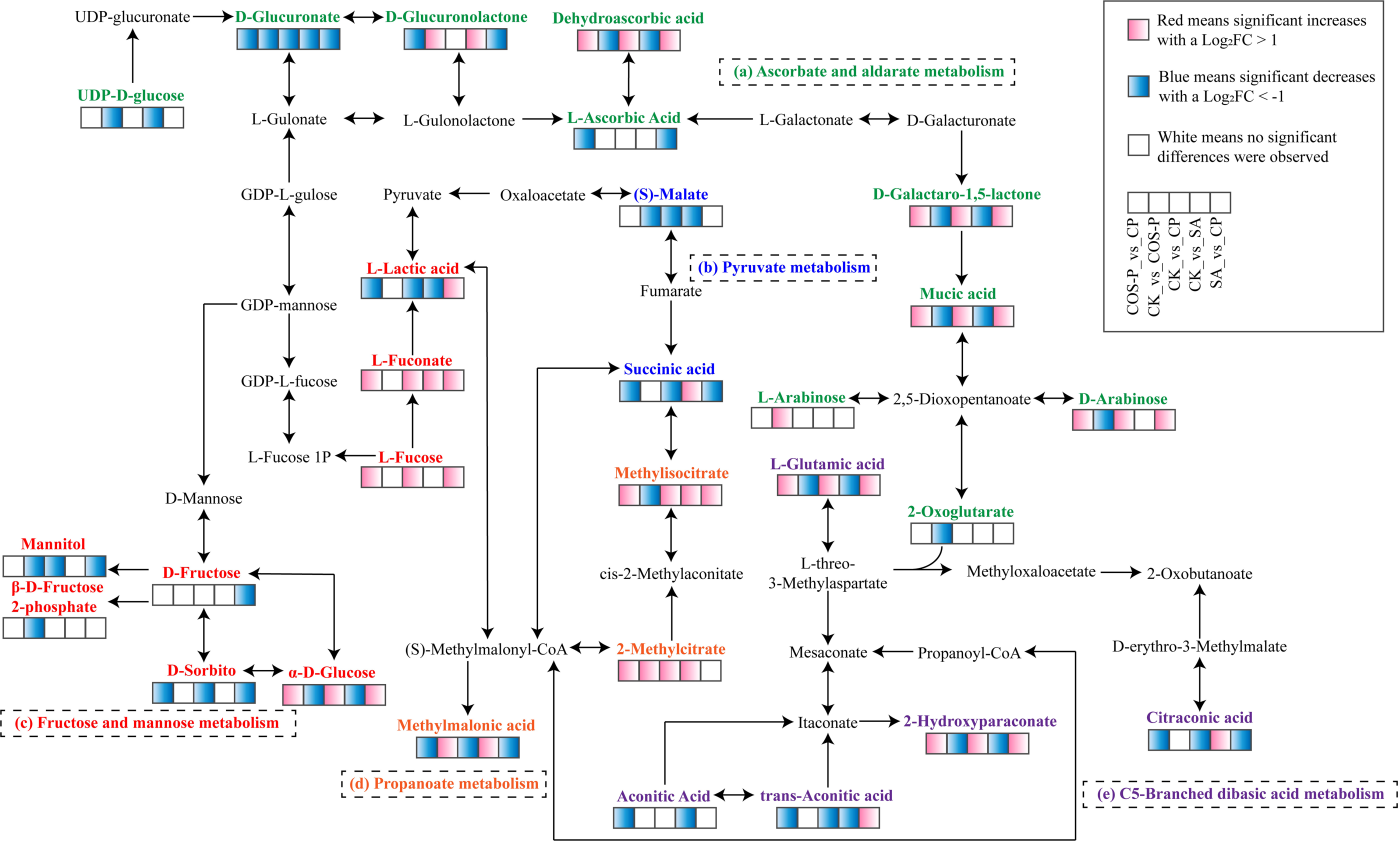
Network of carbohydrate metabolism in cotton seedling leaves.

The carbohydrate metabolism-related KEGG pathways included Ascorbate and aldarate metabolism, Pyruvate metabolism, C5-Branched dibasic acid metabolism, Propanoate metabolism, and Fructose and mannose metabolism (**Supplementary Figure S1**). In Pyruvate metabolism pathway, the Succinic Acid abundance was up-regulated in the SA treatment and that of (S)-Malate was down-regulated, compared with those of CK. The Succinic Acid abundance was down-regulated in the CP treatment compared to SA treatment. In Propanoate metabolism pathway, the abundances of Methylmalonic Acid, Methylisocitrate, and 2-Methylcitrate of the SA treatment were up-regulated compared with those of CK. The Methylmalonic acid abundance was down-regulated in the CP treatment and the Methylisocitrate abundance was up-regulated compared with those of the SA treatment. In Fructose and mannose metabolism pathwa, the abundance of α-D-Glucose of the SA treatment was down-regulated and that of L-Fuconate was up-regulated compared with those of CK. The abundances of α-D-Glucose, L-Fucose, and L-Fuconate of the CP treatment were up-regulated, and those of D-Sorbitol, Mannitol, and D-Fructose were down-regulated compared to SA. In C5-Branched dibasic acid metabolism pathway, the Citraconic Acid abundance was up-regulated in the SA treatment but the Aconitic Acid, L-Glutamic Acid, Trans-Aconitic Acid, and 2-Hydroxyparaconate abundances were down-regulated compared with those of CK. The Citraconic Acid abundance was down-regulated in the CP treatment but the L-Glutamic Acid, Trans-Aconitic Acid, and 2-Hydroxyparaconate abundances were up-regulated compared to SA treatment. In Ascorbate and aldarate metabolism pathway, the abundance of D-Glucuronolactone was up-regulated in the SA treatment, but D-Glucuronate, Mucic Acid, Dehydroascorbic acid, and D-Galactaro-1,5-lactone abundances were down-regulated compared with those of CK. The abundances of D-Glucuronate, D-Glucuronolactone, and L-Ascorbic acid of the CP treatment were down-regulated, and the abundances of D-Arabinose, Mucic Acid, Dehydroascorbic acid, and D-Galactaro-1,5-lactone were up-regulated compared to SA treatment (**Figure 5**).

### 3.5 Correlations among cotton seedling growth indexes, ion content, photosynthetic characteristics, and DAMs

**Figure 6:**
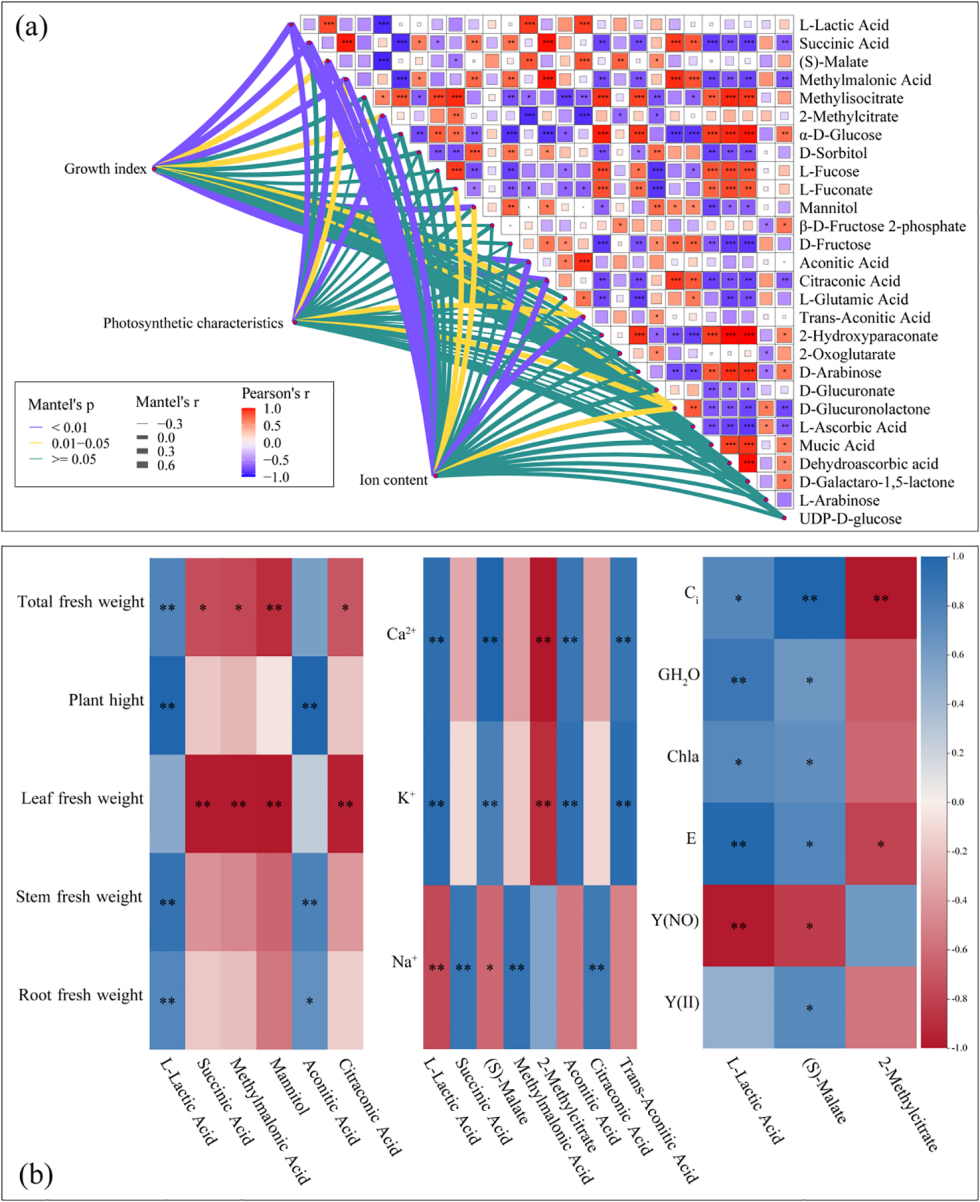
Relationships between differentially abundant metabolites (DAMs), growth indexes, photosynthetic characteristics, and ion content in cotton seedling leaves **(a)**. Correlation analysis between DAMs, growth indexes, and physiological indicators of cotton seedlings leaves **(b)**. Correlation between the metabolites and each variable was tested by the Mantel test. Line width represents the distance-dependent Mantel’r statistic. Purple lines indicate significant differences at *p< 0.01*, yellow lines indicate significant differences at *p< 0.01*, and green lines indicate insignificant differences. Pearson’s correlation coefficients are indicated by shades of color. *, *p< 0.05*; **, *p< 0.01*; ***, *p< 0.001*.

There was a correlation between the growth indexes of cotton seedlings and the abundances of leaf metabolites (L-Lactic Acid, Succinic Acid, Methylmalonic Acid, Mannitol, Aconitic Acid, and Citraconic Acid) (Mantel’s, *p<* 0.01) (**Figure 6a**). The plant height and stem fresh weight had a negative correlation (*p<* 0.01) with L-Lactic Acid and Aconitic Acid abundances. The leaf fresh weight had a positive correlation (*p<* 0.01) with Succinic Acid, Methylmalonic Acid, Mannitol, as well as Citraconic Acid abundances. The root fresh weight had a positive correlation (*p<* 0.01) with L-Lactic Acid abundance. The total fresh weight had a positive correlation (*p<* 0.01) with L-Lactic Acid abundance, and a negative correlation with the abundance of Mannitol (**Figure 6b**). Ion content was correlated with the abundances of L-lactic acid, Succinic acid, (S)-Malate, Methylmalonic acid, 2-methylcitrate, Aconitic acid, Citraconic acid, and Trans-Aconitic acid (Mantel’s, *p<* 0.01) (**Figure 6a**). Leaf K^+^ and Ca^2+^ contents had a positive correlation (*p<* 0.01) with L-Lactic Acid, (S)-Malate Aconitic Acid, and Trans-Aconitic Acid abundances, and a negative correlation (*p<* 0.01) with 2-Methylcitrate abundance. Leaf Na^2+^ content had a positive correlation (*p<* 0.01) with Succinic Acid, Methylmalonic Acid, as well as Citraconic Acid abundances, and a negative correlation (*p<* 0.01) with L-Lactic Acid abundance (**Figure 6b**).

Leaf photosynthetic characteristics was correlated (Mantel’s, *p<* 0.01) with the abundances of L-Lactic Acid, (S)-Malate, as well as 2-Methylcitrate (**Figure 6a**). The leaf *Ci* had a positive correlation (*p<* 0.01) with (S)-Malate abundance, and a negative correlation (*p<* 0.01) with 2-Methylcitrate abundance. Leaf *G_H_2_O* and *E* had a positive correlation (*p<* 0.01) with L-Lactic acid. abundance Leaf *Y(NO)* had a negative correlation (*p<* 0.01) with L-Lactic acid abundance (**Figure 6b**).

## 4 Discussion

Soil salinization can disrupt the plasma membrane selective permeability as well as the cytoplasmic membrane of plants, interfere with various metabolic processes, and cause osmotic stress and nutrient deficiency. This leads to plant physiological and biochemical metabolism disorders, inhibiting plant growth and development (Kumari et al., 2025). In the present research, salt stress significantly decreased the cotton leaf, stem, root fresh weight, and plant height compared to the CK (**Figure 1**). This is the same as Ibrahim et al. (2017). The growth status and biomass accumulation of plants can intuitively reflect the level of salt stress-induced damages to crops as well as plant resistance to stress. Salt stress directly inhibits crop vegetative growth and root nutrient absorption, inhibiting biomass accumulation and ultimately reducing crop yield (Zhao et al., 2023). In this research, 10 g·L^-1^ of salt stress decreased the, chlorophyll fluorescence parameters, photosynthetic pigment contents, as well as photosynthetic rate of cotton seedling leaves compared with the CK (**Figure 2**). This may be due to the fact that the ultrastructure of chloroplasts changes significantly under salt stress, causing the deformation of chloroplasts, unclear chloroplast membrane, and loose and disintegrated thylakoid layer (Yu et al., 2022). In addition, many studies have reported that chitosan could enhance plants’ tolerance to abiotic stresses by repairing plant photosystem II, reducing oxidative damage, and maintaining photosynthetic pigments (Shehzad et al., 2020; Zong et al., 2017; Zou et al., 2016). This research found that tCOS-PA increased leaf, stem, root fresh weight and photosynthetic rate of salt-stressed cotton seedlings compared with CK (**Figure 2**). This may be due to the fact that chito-oligosaccharide has active groups such as -NH_2_ and -OH and is easily soluble, highly-antibacterial, highly-anti-inflammatory, and highly-antioxidant. Besides, chito-oligosaccharides can form chito-oligosaccharide complexes with metal ions, proteins, polysaccharides, lipids, flavonoids, etc. through complexation, electrostatic interaction, aminotransferation, Maillard reaction, chito-oligosaccharide amidation, esterification, carboxylation and other methods, increasing the absorption of essential mineral elements by plants (Liu et al., 2022).

The normal growth and metabolism of plants require an ion homeostasis within cells. In the present research, the leaf Na^+^ content grew sharply and leaf K^+^ content decreased under salt stress compared with those of CK (**Figure 1c**). This indicates that salt stress disrupts the ion homeostasis in plants. Salt stress affects ion distribution in plant cells to disrupt ion homeostasis, resulting in a large amount of Na^+^ accumulation and inhibited K^+^ absorption. This further causes ionic toxicity as well as antagonism, inhibiting crop growth & development (Joshi et al., 2022). After the application of COS-PA, the Ca^2+^ and K^+^ contents of cotton seedlings were enhanced, but the content of Na^+^ was reduced, thereby increasing K^+^/Na^+^ ratio (**Figure 1c**). This helps maintain the normal physiological function of cotton seedlings, and promote Na^+^ efflux, reducing the damage of salts to plant tissues. The increase of Ca^2+^ content can also help cotton seedlings maintain the stability of membrane-bound proteins, cell membrane, cell wall, and intracellular homeostasis, reducing the negative impacts of ion toxicity and secondary stresses (Han et al., 2014).

Plants are subjected to ionic stress under salt stress, causing physiological & metabolic disorders, inhibited photosynthesis and respiration, and changed metabolites (Du et al., 2021). Primary metabolites respond significantly to stresses under limited CO_2_ assimilation (Wang et al., 2019). Organic acids are plant photosynthesis’s intermediate products and metabolically active solutes that maintain cation balance (Medeiros et al., 2021), reflecting crops’ surviving and basal metabolism-maintaining ability (Samanta et al., 2020). Besides, organic acids drive proton & electron transfer (Medeiros et al., 2021), providing energy through proton gradients and contributing to the redox shuttling between cellular compartments. In this study, five carbohydrate metabolism pathways (Ascorbate and aldarate metabolism, C5-Branched dibasicacid metabolism, Pyruvate metabolism, Propanoate metabolism, and Fructose and mannose metabolism) were significantly activated in cotton seedlings under the joint impacts of salt stress and COS-PA (**Figure 5**). These five pathways are the main carbon metabolism components, and their metabolic activities are closely associated with the photosynthesis-based carbon sequestration capacity of seedlings (Zhao et al., 2020). In these pathways, the abundance of L-lactic acid, a related intermediate product, was significantly down-regulated under salt stress (**Figure 6**). Besides, its abundance had a positive correlation with leaf *G_H_2_O* and *E* and a negative correlation with leaf *Y(NO)* (*p<* 0.01). This indicates that salt stress decreases energy production as well as inhibits cotton growth & development (Rajkumari et al., 2023). This is confirmed by Lu et al. (2022), that is, the content of some organic acids in grapevine decreased due to that salt stress and alkali stress inhibits photosynthesis and causes the lack of carbon source. However, Kang et al. (2022) reported that nanoselenium application stimulated plant photosynthesis by promoting short-wave light absorption, affecting carbohydrate metabolism. In this study, COS-PA application increased cotton leaf L-Lactic Acid abundance under salt stress; Besides, L-Lactic Acid abundance had a positive correlation with the contents of K^+^ and Ca^2+^ in leaves (*p<* 0.01). This indicates that COS-PA can not only help cotton seedlings absorb some inorganic ionic substances (K^+^, Ca^2+^), but also promote the accumulation of small molecules, which work together to reduce intracellular water potential, maintaining the ion balance and the normal growth of cotton seedlings (Wang et al., 2021a). However, it was also found the up-regulation of the abundances of three key metabolites (Succinic Acid, Methylmalonic Acid, and Citraconic Acid) involved in the five pathways in salt-stressed cotton seedlings. This indicates that C5-Branched dibasic acid metabolism, Propanoate metabolism, and Pyruvate metabolism pathways could be activated, and Succinic Acid, Methylmalonic Acid, Citraconic Acid in cotton seedlings were the main organic solutes against stress under salt stress to maintain ion balance (K^+^, Ca^2+^, Na^+^) and alleviate salt stress (Yan et al., 2022). These are the same as the results of Yan et al. (2022), i.e., the significant increase of organic acid content in *Leymus chinensis* seedlings under alkaline stress. Besides, three key metabolites (Succinic acid, Methylmalonic acid, and Citraconic acid)also had a positive correlation (*p<* 0.01) with the content of leaf Na^+^. This indicates that these primary metabolites could alleviate the cellular ion imbalance induced by excess adsorption of Na^+^ by crops under salt stress, maintaining osmotic balance between plant cytoplasm and external environment (Rajkumari et al., 2023; Li et al., 2021). Steuer et al. (2007) stated that succinic acid is a vital constituent of the citric acid cycle and it helps in the production of energy through respiration. This process is beneficial for maintaining the nutritional balance of plants under unfavorable environment, and it exerts a positive influence on enhancing the salt tolerance of cotton, as well as on its respiration and photosynthesis. In this study, COS-PA application increased L-Lactic Acid abundance under salt stress (**Figure 5**). L-Lactic Acid can reduce the accumulation of salt ions (Na^+^, K^+^) in the soil, thereby maintaining the osmotic balance of plant roots. This, in turn, alleviates the detrimental effects of salt stress on plants and promotes plant growth. Moreover, L-Lactic Acid can regulate the acid-base balance in the soil. It increases the content of soluble phosphorus in the soil, providing a suitable environment for rhizosphere microorganisms. This provides essential mineral nutrients for promoting plant growth (Chen et al., 2023).

## 5 Conclusion

Chito-oligosaccharide modifier COS-PA alleviated the salt stress damages to ion homeostasis, metabolism, photosynthesis, as well as growth of cotton seedlings. The toxicity of salts on cotton seedlings was mainly manifested in the decreases in fresh weight & plant height; The ion toxicity was mainly manifested in an growth in Na^+^ content as well as a decrease in K^+^ and Ca^2+^ contents. COS-PA application alleviated salt stress-induced toxicity by enhancing photosynthesis and carbohydrate metabolism in seedling leaves. Especially, cotton leaf L-Lactic Acid abundance was reduced under salt stress, but it was increased after COS-PA application. The increased L-Lactic Acid abundance affected the Na^+^ content, Ca^2+^ content, fresh weight of each part, stomatal conductance, chlorophyll concentration (Chl a), transpiration rate, as well as actual quantum yield of PS II. In addition, salt stress led to increased abundances of Succinic Acid, Methylmalonic Acid, and Citraconic Acid compared with CK, which further reduced the fresh weight of leaves as well as increased Na^+^ content. However, COS-PA application enhanced leaf fresh weight and decreased the Na^+^ content of cotton seedlings by reducing their abundances. The application of COS-PA also enabled more metabolites to participate in crops’ salt stress responses, which improved the salt stress tolerance. This study provides a feasible method for achieving the sustainable agricultural development on salinized lands.

## Data Availability

The original contributions presented in the study are included in the article/**Supplementary Material**. Further inquiries can be directed to the corresponding authors.
