# Correction: Chito-oligosaccharide composites enhanced the adaptability of cotton seedlings to salinized soil by modulating photosynthetic efficiency and metabolite

**DOI:** 10.3389/fpls.2025.1668787

**Published:** 2025-09-09

**Authors:** Mengjie An, Linlin Zhang, Qianqian Wang, Kaidi Ren, Qinjuan Wang, Dongmei Lin, Yongqi Zhu, Yonghong Fan

**Affiliations:** Xinjiang Key Laboratory of Biological Resources and Genetic Engineering, College of Life Science & Technology, Xinjiang University, Urumqi, Xinjiang, China

**Keywords:** composite soil amendment, salt stress, ion content, photosynthesis, carbohydrate metabolism

In the published article, there was an error in the **Funding** statement. “This work was supported by the Natural Science Foundation of Xinjiang Uygur Autonomous Region (2023D01C16), the “Tianchi Talents” Introduction Plan Project of Xinjiang Uygur Autonomous Region (510523005128) and the Science and Technology Plan Projects of Xinjiang Uygur Autonomous Region (202206120029).” The correct **Funding** statement appears below.

“This work was supported by the Key R&D Program Project of Xinjiang Uygur Autonomous Region of China (2022B02053-2), the Natural Science Foundation of Xinjiang Uygur Autonomous Region(2023D01C16).”

The original version of this article has been updated.

