# Supplementary figures and images for: Chito-oligosaccharide composites enhanced the adaptability of cotton seedlings to salinized soil by modulating photosynthetic efficiency and metabolite
Source: Front Plant Sci. 2025 Jul 4;16:1615321. doi: 10.3389/fpls.2025.1615321 (PMC12271869; doi:10.3389/fpls.2025.1615321)

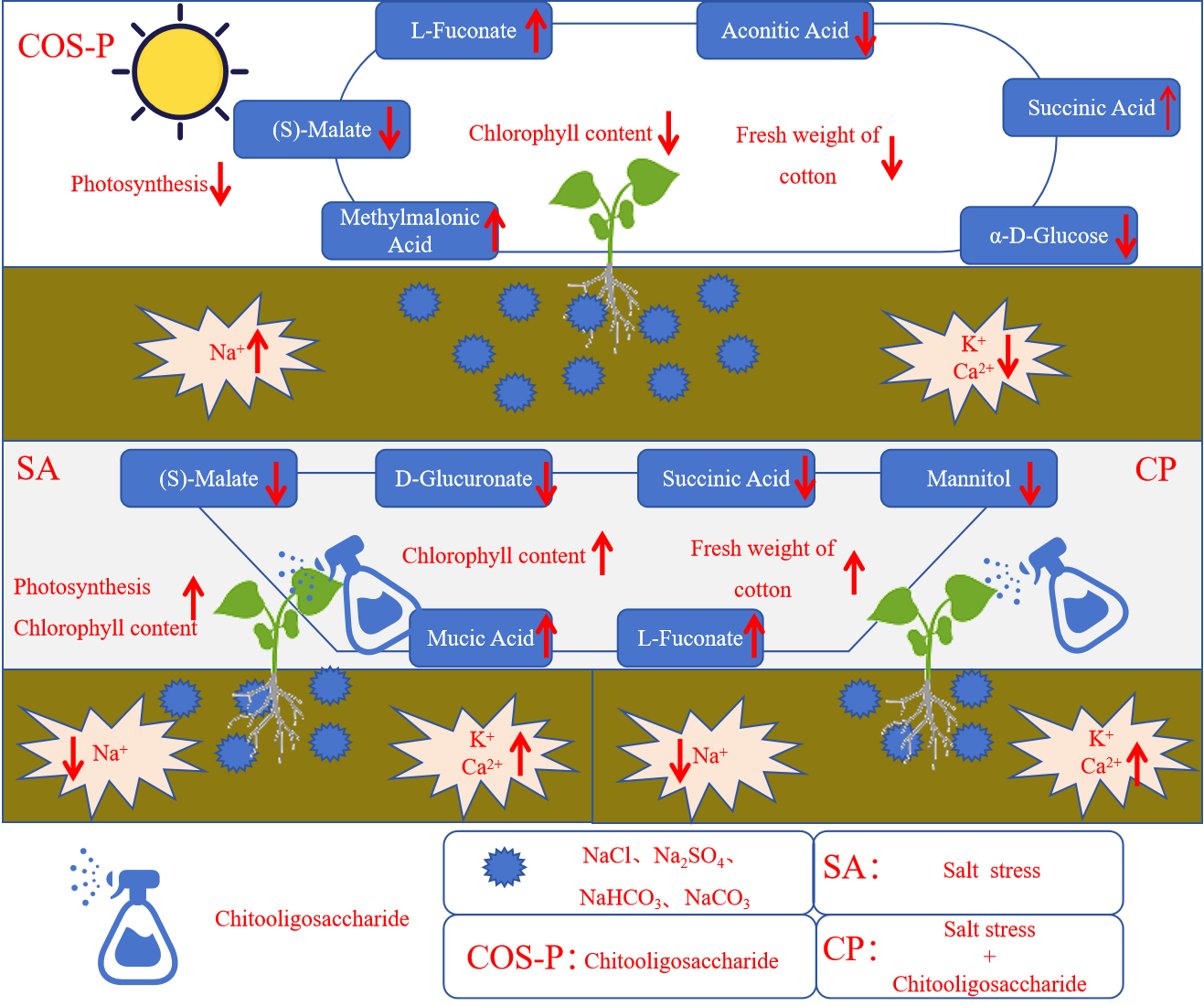

Supplement: Supplementary file 1 [file Image1.tif]
