# Supplementary material for: Chito-oligosaccharide composites enhanced the adaptability of cotton seedlings to salinized soil by modulating photosynthetic efficiency and metabolite
Source: Front Plant Sci. 2025 Jul 4;16:1615321. doi: 10.3389/fpls.2025.1615321 (PMC12271869; doi:10.3389/fpls.2025.1615321)
Supplement: Supplementary file 2 [file DataSheet1.doc]

**
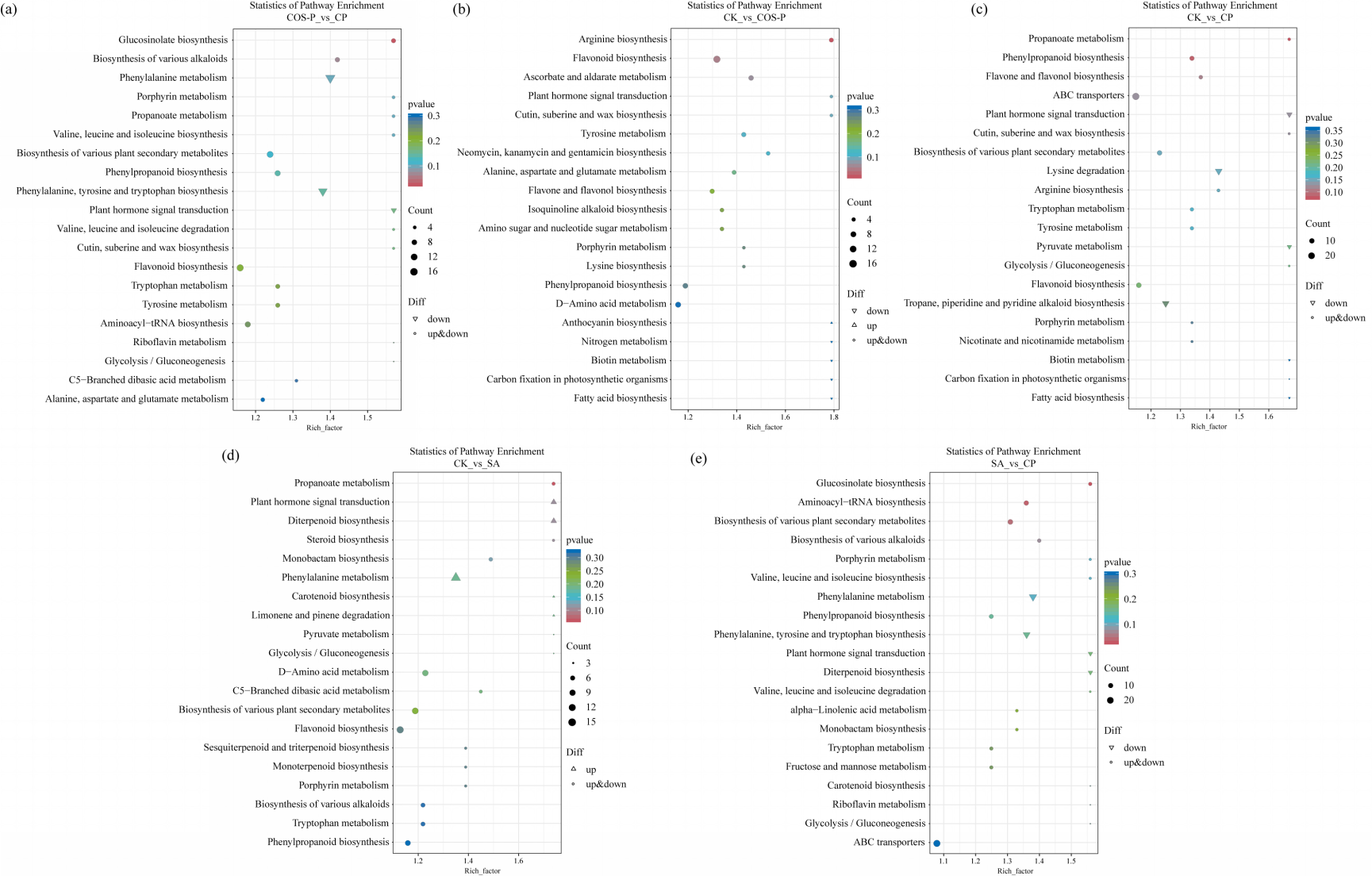
**

**Fig. S1.** Key metabolic pathways in cotton leaves under different treatments. The degree of enrichment is evaluated by Rich_factor, p-value, and number of metabolites enriched in each pathway.
